# Lung microbiome on admission in critically ill patients with acute bacterial and viral pneumonia

**DOI:** 10.1038/s41598-023-45007-4

**Published:** 2023-10-18

**Authors:** Jose María Marimón, Ane Sorarrain, Maria Ercibengoa, Nekane Azcue, Marta Alonso, Loreto Vidaur

**Affiliations:** 1 Biodonostia, Infectious Diseases Area, Respiratory Infection and Antimicrobial Resistance Group, Microbiology Department, Osakidetza Basque Health Service, Donostialdea Integrated Health Organization, 20014 Donostia-San Sebastian, Spain; 2 Microbiology Department, Donostia University Hospital, 20014 Donostia-San Sebastián, Spain; 3 Intensive Care Unit, Donostia University Hospital, 20014 Donostia-San Sebastián, Spain; 4 Centro de Investigacion Biomedica en Red de Enfermedades Respiratorias (CIBERES), Instituto de Salud Carlos III, Madrid, Spain

**Keywords:** Clinical microbiology, Microbial communities, Infectious-disease diagnostics, Molecular medicine

## Abstract

Composition of pulmonary microbiome of patients with severe pneumonia is poorly known. The aim of this work was to analyse the lung microbiome of patients admitted to the intensive care unit  (ICU) with severe community acquired pneumonia (CAP) between 2019 and 2021 in comparison with a control group of 6 patients undergoing digestive surgery. As a second objective, the diagnostic capabilities of metagenomics was also studied in a small group of selected patients. The lung microbiome of patients with viral (5 with Influenza A and 8 with SARS-CoV-2) pneumonia at admission showed a similar diversity as the control group (p = 0.140 and p = 0.213 respectively). Contrarily, the group of 12 patients with pneumococcal pneumonia showed a significant lower Simpson´s index (p = 0.002). In the control group (n = 6) Proteobacteria (36.6%), Firmicutes (24.2%) and Actinobacteria (23.0%) were the predominant phyla. In SARS-CoV-2 patients (n = 8), there was a predominance of Proteobacteria (mean 41.6%) (*Moraxella* and *Pelomonas* at the genus level), Actinobacteria (24.6%) (*Microbacterium*) and Firmicutes (22.8%) mainly *Streptococcus*, *Staphylococcus* and *Veillonella*. In patients with Influenza A pneumonia (n = 5) there was a predominance of Firmicutes (35.1%) mainly *Streptococcus* followed by Proteobacteria (29.2%) (*Moraxella, Acinetobacter* and *Pelomonas*). In the group of pneumococcal pneumonia (n = 12) two phyla predominated: Firmicutes (53.1%) (*Streptococcus*) and Proteobacteria (36.5%) (*Haemophilus*). In the 7 patients with non-pneumococcal bacterial pneumonia *Haemophilus influenzae* (n = 2), *Legionella pneumophila* (n = 2), *Klebsiella pneumoniae*, *Streptococcus pyogenes* and *Leptospira* were detected by metagenomics, confirming the diagnosis done using conventional microbiological techniques. The diversity of the respiratory microbiome in patients with severe viral pneumonia at ICU admission was similar to that of the control group. Contrarily, patients with pneumococcal pneumonia showed a lower grade of diversity. At initial stages of SARS-CoV-2 infection, no important alterations in the pulmonary microbiome were observed. The analysis of bacterial microbiome showed promising results as a diagnostic tool.

## Introduction

One of the most frequent community acquired infections requiring hospitalization is pneumonia, which in severe cases requires admission into the intensive care units (ICU) and mechanical ventilation^1^. Despite early antimicrobial treatment and support measures, mortality due to severe pneumonia is still very high and new approaches in respiratory therapy are being sought to try to improve their outcomes^2^. Among the new research in pneumonia, the study of the role of the lung microbiome has been pointed out as a new key therapeutic target for the prevention and treatment of critical respiratory illness^3^. Although the lung microbiome is composed of bacterial, viral and fungal populations, most studies are based on the analysis of the bacteriome, in which all the bacterial 16S rRNA genes present in the sample are amplified and sequenced using next-generation sequencing (NGS). This technology has also been used with diagnostic purposes, as a high number of pneumonias remain without etiologic diagnosis despite efforts in detecting the responsible pathogen^1,4,5^

The lung microbiota in healthy individuals is conceptualized not as a static entity but as a dynamic balance between microbial immigration, clearance and replication under the strict alveolar conditions^6,7^. It has been demonstrated that bacterial communities of the healthy lung are quite similar to those found in the mouth but at lower concentrations, lower diversity and different composition^8^. In fact, the lung microbiome of each person seems to be unique^9^. In this model, pneumonia causative agents will alter the previous homeostasis producing decrease in biodiversity, increase in the microbial biomass and inflammation in the host^7^. However, and mainly due to the lack of samples from the lower-respiratory tract of patients before developing pneumonia, it is quite difficult to determine whether differences in the microbiota are a cause or a consequence of pulmonary infections^10^.

Many studies on respiratory microbiome have been done, most of them in patients with chronic pulmonary diseases as asthma, chronic obstructive pulmonary disease (COPD), cystic fibrosis or receiving mechanical ventilation^11,12^. However, the number of studies focused on the lung microbiome in community acquired pneumonia (CAP) are scant, probably due to the great number of challenges that the study of the lung microbiome poses. Among them, the invasiveness of obtaining samples via bronchoalveolar lavages (BAL), the frequent use of antibiotics before sampling which affects the bacterial composition, or the role of contamination of reagents used in amplification and sequencing techniques are the most important^13^.

In this work we analyse the lung microbiome of 31 patients admitted to the ICU due to severe bacterial and/or viral pneumonia and compare it with the lung microbiome of 6 control patients submitted to digestive surgery. All samples were taken by protected mini-BAL in the first 24 h since admission, and the use of antibiotics was limited to 48 h to avoid antibiotic treatment interferences in the microbiome composition. As no samples previous to the development of pneumonia were available, only description of the microbiome found at admission was analysed. As a secondary aim, the utility of metagenomic NGS (mNGS) as a microbiological diagnostic method for bacterial pneumonia was studied in the non-protected BAL of a subset of 7 selected patients with non-pneumococcal bacterial pneumonia.

## Methods

### Subjects

Between January 2019 and May 2021, 31 adult patients with severe CAP admitted to the ICU were prospectively enrolled in the study (Table 1). The including criteria were: (1) Adult patients admitted with clinical and radiological criteria of severe CAP according to IDSA/ATS guidelines^14^, (2) BAL samples collected in the first 24 h since admission to the ICU, (3) No antibiotic treatment for more than 48 h. Of the 31 patients of whom respiratory microbiome was investigated, 7 have not received antibiotics before obtaining the mini-BAL sample (6 of them with SARS-CoV-2 pneumonia), 9 had received antibiotics during the previous 24 h, and 15 had received antibiotics in the previous 48 h (Table 1). As control group, 6 postsurgical intubated patients due to gastrointestinal disease were also analysed. Pulmonary samples were collected using a protected blind mini-BAL (Combicath™ Plastimed, France) and immediately taken to the microbiology department.

**Table 1:** Patients included in the study of the respiratory microbiome according to the etiology of pneumonia.

Group	Description		n	Average age^1^ (range)	Females/Males	Patients (n) with previous antibiotic treatment^2^		
						No	< 24 h	< 48 h
1	Control group^3^		6	67.5 (58–76)	1/5	5	1	0
2a	Viral^4^	Influenza AH1 virus	5	55 (36–76)	1/4	0	1	4
		Rhinovirus	3	68 (64–73)	1/2	0	1	2
		Respiratory syncytial virus (RSV)	1	77	0/1	0	0	1
2b	SARS-CoV-2 virus		8	61.4 (42–71)	2/6	6	2	0
2c	*Streptococcus pneumoniae*		12	62.6 (37–76)	4/8	0	5	7
2d	Non-etiological diagnosis		2	79–84	1/1	1	0	1

Days of previous antibiotic treatment prior to admission in ICU are also described.

^1^Age in years.

^2^No = no previous antibiotic treatment; < 24 h: antibiotic treatment in the 24 h prior to admission; < 48 h: antibiotic treatment in the 48 h prior to admission.

^3^Non-pneumonic patients undergoing digestive surgery.

^4^Others than SARS-CoV-2 pneumonia.

Apart from the study of the lung microbiome, the non-protected BAL of 7 patients diagnosed with non-pneumococcal bacterial pneumonia were arbitrarily selected, based on the pathogen detected by microbiological culture or other conventional methods, to study the possible utility of mNGS as a diagnostic method for bacterial pneumonia.

### Microbiological procedures

Microbiological analysis for the etiological diagnosis of pneumonia included blood-culture (BD BACTEC™ blood culture systems), urine antigen test for *Streptococcus pneumoniae* and *Legionella pneumophila* detection (Sofia FIA^®^ Quidel Corporation, San Diego, CA, USA), and qRT-PCR targeting viral respiratory pathogens (Allplex™ Respiratory Panels 1, 2 and 3, Seegene, South Korea). For other less frequent bacterial pathogens causing pneumonia (*Mycoplasma pneumoniae* and *Chlamydophila pneumoniae*) a PCR of a pharyngeal swab was performed according to manufactures instructions (CerTest Biotec SL, Spain).

In patients with diagnosis of SARS-CoV-2 pneumonia, diagnosis of SARS-CoV-2 had been commonly done some days before admission using commercial RT-PCR (Allplex SARS-CoV-2 assay, Seegene, South Korea) on nasopharyngeal swabs. Alpha (lineage B.1.1.7) was the predominant SARS-CoV-2 variant present when samples of these patients were collected.

### Microbiome analysis

Nucleic acids from 1 mL of the mini-BAL were extracted using the NUCLISENS easyMAG platform (bioMérieux) according to manufacturer´s recommendations and eluted in a final volume of 100 µl of elution buffer.

Microbiome was determined using the Ion 16S Metagenomics Kit (Thermo Fisher Scientific) that amplifies 7 hypervariable regions (V2, 3, 4, 6–7, 8, 9) of the 16S rRNA gene in two amplification reactions (primer sets V2-4-8 and V3-6,7–9, respectively) with the following PCR conditions: 95 °C for 10 min, 30 cycles at 95 °C for 30 s, 58 °C for 30 s, and 72 °C for 20 s, followed by 72 °C for 7 min. After amplification, samples were run in an agarose gel and those not showing amplification were subjected to a nested PCR using the same primers and PCR conditions.

The nested PCR was performed on 6 samples that had amplified in the initial PCR, and the sequencing results of the first (standard) and nested PCRs were compared to verify if there were differences in the composition of the microbiome between both PCRs as nested PCR was performed on BAL samples with low bacterial load.

Resulting PCR products of each sample were mixed and cleaned up using the Sera-Mag Select magnetic beads (Cytiva, Sheffield, UK). Purified amplicons were quantified using the Qubit Fluorometer kit (Invitrogen, Carlsbad, CA, USA). Libraries were prepared using the Ion Plus fragment Library Kit. Briefly, amplicons were end repaired and purified before adapters and barcodes were ligated and nick repaired using DNA Ligase and nick repair polymerases. Libraries were cleaned using the Sera-Mag Select Reagent and quantified by qRT-PCR. Finally, samples were combined in equimolar concentrations and sequenced with the commercially available ION PGM™ Sequencing 400 kit on an ION PGM™ System (Thermo Fisher Scientific, Waltham, MA, USA), using an Ion 318™ Chip v2, with a maximum of 12 samples per chip.

Data was analysed with the Ion Reporter software 5.18.2 using the consensus data of all variable regions. Alpha diversity results were produced using the QIIME’s open-source bioinformatics pipeline. Alpha diversity was calculated using the Simpson and Shannon diversity indexes.

### Utility of metagenomic Next-Generation Sequencing (mNGS) for etiological diagnosis of patients with CAP

The same mNGS technique used in the study of the microbiome was applied to the BAL of 7 patients with radiological and clinical criteria of severe CAP^14^. These patients were arbitrarily selected according to the etiologic agent found in any of their samples using conventional microbiological diagnostic techniques. In these patients, differing from the protected mini-BAL used in the microbiome analysis, the rest of the BAL used for routine microbiological diagnosis was used for mNGS.

Patients with pneumococcal pneumonia were intentionally excluded from this part of the study as they had been already included in the study of the microbiome of patients with pneumococcal NAC.

### Statistics

Continuous variables were compared using the unpaired t test using the Graph pad Prism 5 software. A p value of < 0.05 was considered as statistically significant.

### Ethics

The study was approved by the regional (Gipuzkoa) Ethics Committee, reference MOZ-NBI-2017–01 and all patients or their legal representatives gave written informed consent to be included in the study. All experiments were performed in accordance with relevant guidelines and regulations.

## Results

### Validation of the nested-PCR

The pulmonary microbiome of six different patients was studied both on direct DNA extraction (standard PCR) and after performing a nested PCR of the first amplified product using the same sets of primers and PCR and sequencing conditions to check possible differences in the results. The total number of reads was bigger in five of the six samples in the standard PCR compared to the nested PCR (Table 2). Almost no differences were observed in the distribution and richness of the most frequent genus in each sample (Supplementary Table S1).

**Table 2:** Comparison of the results of the microbiome at the genus level of six BAL samples on standard-PCR and nested-PCR amplification of the 16S rRNA gene (only genus with a relative abundance of more than 1% are shown).

	SvsN^a^-1^a^				SvsN-2				SvsN-3				SvsN-4				SvsN-5				SvsN-6			
	Standard		Nested		Standard		Nested		Standard		Nested		Standard		Nested		Standard		Nested		Standard		Nested	
Genus	Reads^b^	%^c^	Reads^b^	%^c^	Reads^b^	%^c^	Reads^b^	%^c^	Reads^b^	%^c^	Reads^b^	%^c^	Reads^b^	%^c^	Reads^b^	%^c^	Reads^b^	%^c^	Reads^b^	%^c^	Reads^b^	%^c^	Reads^b^	%^c^
*Actinomyces*	3206	2.7	1179	2.1																	158	3.3		
*Bifidobacterium*																					1541	3.2	419	1.8
*Campylobacter*			659	1.2																				
*Dialister*																					4179	8.7	1654	7.0
*Fusobacterium*	1916	1.6	715	1.3																	719	15.0	3295	13.9
*Gemella*	1163	1.0																						
*Granulicatella*	3039	2.5																						
*Haemophilus*	2967	2.5	881	1.6									261,495	83.4	104,978	81.8								
*Leclercia*																					635	1.3	451	1.9
*Microbacterium*																					1927	4.0	529	2.2
*Moraxella*					56,601	33.3	42,205	37.4																
*Neisseria*	758	6.3	5274	9.5					174,689	92.1	75,554	72.3												
*Oribacterium*																					705	1.5		
*Prevotella*	6996	5.8	2612	4.7																	10,072	21.0	2833	11.9
*Propionibacterium*																					143	3.0	694	2.9
*Pseudomonas*																	166,480	98.4	174,901	98.6				
*Rothia*	4530	3.8	1756	3.2																				
*Streptococcus*	74,706	62.2	31,177	56.4	112,972	66.5	70,002	62.0	14,147	7.5	24,273	23.2	33,606	10.7	14,286	11.1					1055	2.2		
*Treponema*																					149	3.1	802	3.4
*Veillonella*	4628	3.9	1461	2.6																	1035	2.2		
Other genus	4150	3.5	1945	3.5	127	0.1	16	0.0	381	0.2	1463	1.4	1086	0.3	156	0.1	1235	0.7	1,677	0.9	7832	16.3	2605	11.0
Non assigned^d^	5290	4.4	7588	13.7	116	0.1	612	0.5	435	0.2	3211	3.1	17,371	5.5	885	6.9	1419	0.8	814	0.5	737	15.3	10,47	44.1
Total	120,171	100	55,247	100	169,816	100	112,835	100	189,652	100	104,501	100	313,558	100	128,27	100	167,715	100	176,578	100	30,314	100	13,282	100

^a^*SvsN* Standard-PCR versus Nested-PCR.

^b^Reads count.

^c^Percentage of total reads.

^d^Non assigned reads.

### Microbiome analysis

#### Lung microbiome in the control group

The lung microbiome was studied in the BAL samples of 6 patients undergoing mechanical ventilation before digestive surgery (Table 3). The mini-BAL samples were obtained when the patients were intubated, just before antibiotic prophylaxis was administered and surgery began. On mean, Proteobacteria (36.6%) was the dominant phylum followed by Firmicutes (24.2%) and Actinobacteria (23%) (Table 4). The number of different phyla in each sample varied between 6 and 13.

**Table 3:** Clinical characteristics of the patients of the control group.

Control	Age/Sex	Digestive pathology	Surgery	Length of surgery	Hemodynamic consequences	Gut mobilization
1	58/M	*Hepatocellular carcinoma*	Hepatectomy	4 h 30 min	Hypotension after surgery	No
2	63/F	*Cholelithiasis*	Cholecystectomy	2 h 30 min	No	No
3	75/M	*Malignant neoplasm of ampulla of Vater*	Pancreatoduodenectomy	7 h 45 min	No	No
4	62/M	Neoplasm of rectosigmoid junction	Laparoscopic sigmoidoscopy	4 h 15 min	No	Yes^1^
5	71/M	Pseudomyxoma	Peritonectomy and hyperthermic intraperitoneal chemotherapy (HIPEC)	8 h 30 min	No	No
6	76/M	Pancreatic head neoplasia	Pancreatoduodenectomy	6 h 20 min	Hypotension during surgery	No

^1^Metronidazole-neomycin intestinal preparation.

**Table 4:** Lung microbiome in the control group at the phylum level.

Phylum	CONTROL-01		CONTROL-02		CONTROL-03		CONTROL-04		CONTROL-05		CONTROL-06		Mean
	Reads^a^	%^b^	Reads^a^	%^b^	Reads^a^	%^b^	Reads^a^	%^b^	Reads^a^	%^b^	Reads^a^	%^b^	%^c^
Acidobacteria											103	0.5	0.1
Actinobacteria	3034	7.6	26,144	51.9	14,033	23.4	21,934	15.3	8004	20.5	4130	19.4	23.0
Armatimonadetes											91	0.4	0.1
Bacteroidetes	6887	17.1	781	1.6	4330	7.2	1943	1.4	2541	6.5	1443	6.8	6.8
Chlamydiae											26	0.1	0.0
Chloroflexi					127	0.2	23	0.0			261	1.2	0.2
Cyanobacteria			98	0.2	191	0.3	66,339	46.1	245	0.6	41	0.2	7.9
Deinococcus-Thermus			64	0.1							41	0.2	0.1
Firmicutes	13,059	32.5	2128	4.2	17,945	29.9	37,790	26.3	8714	22.3	6365	29.9	24.2
Fusobacteria	1521	3.8			252	0.4	106	0.1	83	0.2	19	0.1	0.8
Nitrospirae							16	0.0					0.0
Planctomycetes									303	0.8	129	0.6	0.2
Proteobacteria	15,486	38.6	21,159	42.0	22,983	38.3	15,597	10.8	19,130	49.0	8650	40.6	36.6
Spirochaetes	14	0.0			168	0.3							0.1
Tenericutes	94	0.2									10	0.0	0.0
Verrucomicrobia	64	0.2					59	0.0					0.0
	40,159	100	50,374	100	60,029	100	143,807	100	39,020	100	21,309	100	100

^a^Reads count.

^b^Percentage of reads.

^c^Mean of percentages.

At the genus level and considering those genera that had more than 1% of the assigned reads in each sample, only the genus *Microbacterium* was detected in the mini-BAL of all the controls, which represented 3.2% of the mean of all the reads (Supplementary Table S2). The genus *Propionibacterium*, *Corynebacterium, Acinetobacter*, *Streptococcus* and *Staphylococcus* were found in 5 of the 6 controls (5.3%, 4.5%, 4.4%, 4.3% and 2.8% of the mean of all reads, respectively). The genus *Enterococcus* and *Enhydrobacter* were found in 4 of the 6 controls (5.9% and 3.4% of the mean of all reads, respectively). All the other genera with more than 1% of reads were found in the microbiome of 3 or less of the 6 controls.

The Shannon index for diversity in the microbiome of controls ranged between 3.423 and 4.847 (mean 4.214 ± 0.581) and the Simpson index ranged between 0.840 and 0.947 (mean 0.902 ± 0.038).

#### Lung microbiome of patients with viral pneumonia (non-SARS-CoV-2)

In the lung microbiome of the 5 patients admitted to the ICU with severe pneumonia caused by the 2009 pandemic influenza A virus subtype H1N1 there was a predominance of Firmicutes (mean 35.1%) mainly *Streptococcus*, *Veillonella* and *Staphylococcus* at the genus level followed by Proteobacteria (mean 29.2%) mainly *Moraxella, Acinetobacter* and *Pelomonas* at the genus level (Table 5 and Supplementary Table S3). The other two highly abundant phyla were Bacteroidetes (mainly *Prevotella* and *Porphyromonas*) and Actinobacteria (mainly *Microbacterium* and *Propionibacterium*) with 17.6% and 15.2% mean abundance, respectively.

**Table 5:** Distribution of the respiratory microbiome in adult patients admitted to ICU with Influenza A virus pneumonia.

Patient	Influenza-1		Influenza-2		Influenza-3		Influenza-4		Influenza-5		Mean
Phylum	Reads^a^	%^b^	Reads^a^	%^b^	Reads^a^	%^b^	Reads^a^	%^b^	Reads^a^	%^b^	%^c^
Actinobacteria	5542	17.1	4397	19.4	840	2.2	20,173	13.7	8252	23.4	15.2
Bacteroidetes	3730	11.5	2904	12.8	2354	6.0	36,739	24.9	11,651	33.0	17.6
Deinococcus-Thermus									75	0.2	0
Firmicutes	11,859	36.6	5479	24.2	20,668	52.9	74,647	50.6	3999	11.3	35.1
Fusobacteria		0.0	337	1.5	2488	6.4	683	0.5	98	0.3	1.7
Proteobacteria	11,258	34.8	8462	37.4	12,668	32.4	15,384	10.4	10,964	31.1	29.2
Spirochaetes			1058	4.7					217	0.6	1.1
Verrucomicrobia					36	0.1					0
Total	32,389	100	22,637	100	39,054	100	147,626	100	35,256	100	100.0

^a^Reads count.

^b^Percentage of reads.

^c^Mean of percentages.

**Figure 1:**
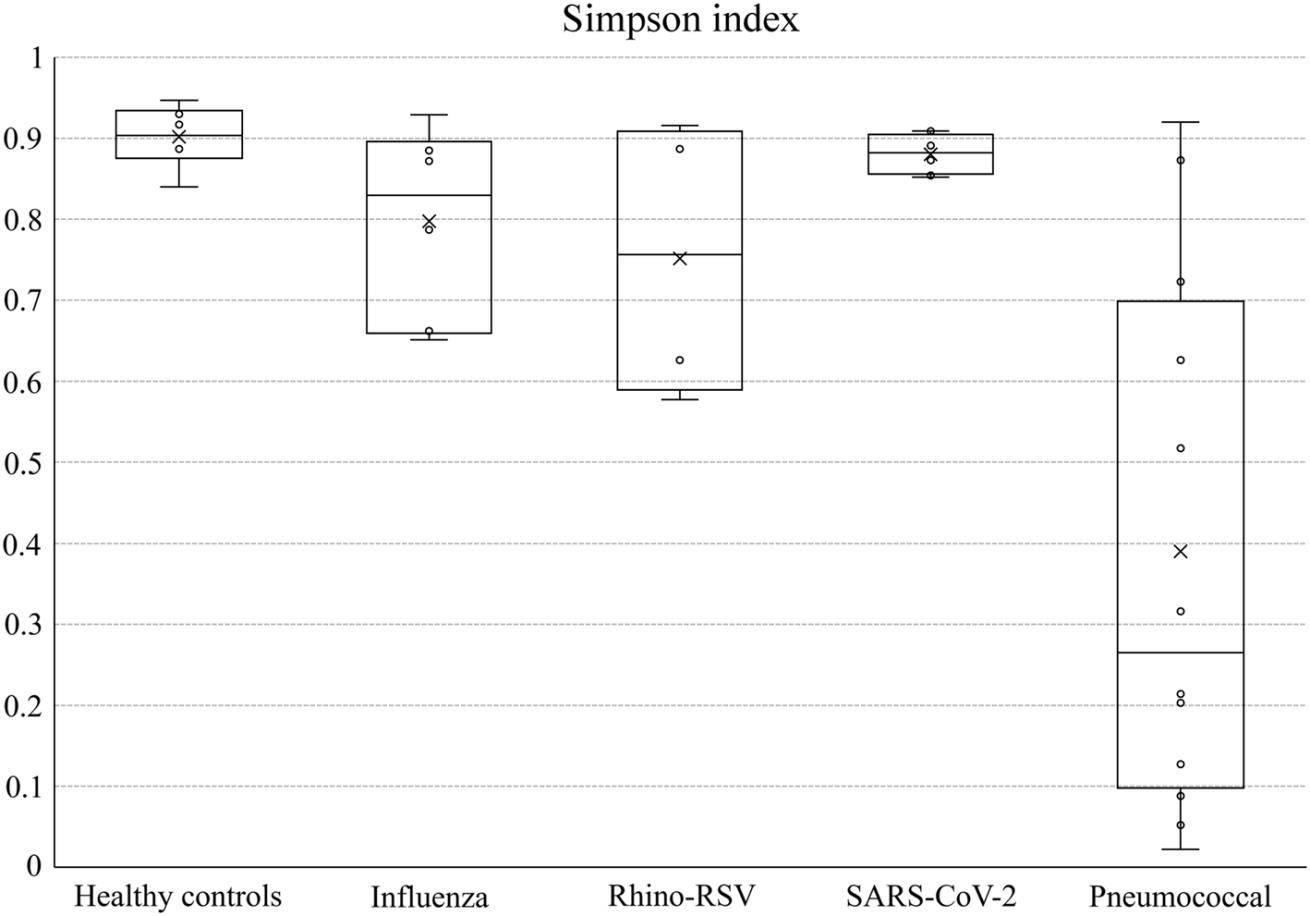
Simpson index showing the microbiome diversity of mini-BAL samples of patients with pneumonia caused by Influenza A virus, SARS-COV-2, Rhinovirus and RSV, and pneumococcal and of control patients without respiratory symptoms.

The Shannon diversity index of the lung microbiome from patients with Influenza virus pneumonia ranged between 2.097 and 4.335 (mean 3.333 ± 0.902). The Simpson diversity index ranged between 0.651 and 0.929 (mean 0.825 ± 0.110) (Fig. 1).

There were also 4 patients in which Rhinovirus (3 patients) or RSV were the only respiratory pathogen detected in any sample. Proteobacteria (mean 62.1%), Firmicutes (22.4%) and Actinobacteria (9.5%) were the most abundant phyla (Supplementary Table S4). At the genus level, *Streptococcus* (17.2%), *Haemophilus* (27.2%) and *Microbacterium* (4.4%) were the most abundant and were found in 4, 3 and 3 of these patients, respectively. The mean Shannon and Simpson diversity indexes of the microbiome of these 4 patients was 3.114 ± 1.171 and 0.752 ± 0.175.

#### Pulmonary microbiome of patients with SARS-CoV-2 pneumonia

In total, we studied 8 patients with SARS-CoV-2 severe pneumonia admitted to the ICU between March 2020 and April 2021, this is, during the first year of the pandemic. The diagnosis of SARS-CoV-2 had been commonly done some days before admission (on mean 3.75 days, range 0–10 days) using a commercial RT-qPCR on nasopharyngeal swabs.

In terms of mean organisms´ abundance in the microbiome of SARS-CoV-2 patients, there was a predominance of Proteobacteria (mean of all samples 41.6%) with *Acinetobacter, Sphingomonas* and *Pelomonas* as predominant genus followed by Actinobacteria (24.6%) mostly *Microbacterium* and *Propionibacterium* and by Firmicutes (22.8%) mainly *Streptococcus* and *Staphylococcus* (Table 6 and Supplementary Table S5).

**Table 6:** Respiratory microbiome composition at the phylum level of patients with severe SARS-CoV-2 pneumonia.

Patient	COVID-1		COVID-2		COVID-3		COVID-4		COVID-5		COVID-6		COVID-7		COVID-8		Mean
Phylum	Reads^a^	%^b^	Reads^a^	%^b^	Reads^a^	%^b^	Reads^a^	%^b^	Reads^a^	%^b^	Reads^a^	%^b^	Reads^a^	%^b^	Reads^a^	%^b^	%^c^
Acidobacteria											322	0.4					0.1
Actinobacteria	14,197	27.6	7619	10.1	12,184	12.5	34,402	28.4	23,529	28.4	18,280	22.8	30,552	31.8	43,955	35.2	24.6
Bacteroidetes	2180	4.2	2340	3.1	16,149	16.6	10,330	8.5	3381	4.1	5029	6.3	3359	3.5	5463	4.4	6.3
Chloroflexi	244	0.5			926	1.0											0.2
Cyanobacteria	279	0.5	1624	2.2	10,191	10.5			212	0.3	6726	8.4	86	0.1	714	0.6	2.8
Deinococcus-Thermus									180	0.2					3584	2.9	0.4
Firmicutes	16,871	32.8	39,784	52.9	30,634	31.5	10,734	8.9	14,763	17.8	10,741	13.4	16,398	17.1	9472	7.6	22.8
Fusobacteria	1689	3.3			10	0.0	1341	1.1	12	0.0			327	0.3			0.6
Gemmatimonadetes			59	0.1	13	0.0											0.0
Lentisphaerae					115	0.1											0.0
Nitrospinae													103	0.1			0.0
Planctomycetes					66	0.1					321	0.4					0.1
Proteobacteria	15,951	31.0	23,342	31.1	26,662	27.5	63,665	52.6	37,939	45.8	38,606	48.2	45,181	47.1	61,581	49.4	41.6
Tenericutes							483	0.4									0.0
Thermotogae			407	0.5	167	0.2			2879	3.5	35	0.0					0.5
Total	51,411	100	75,175	100	97,117	100	120,955	100	82,895	100	80,060	100	96,006	100	124,769	100	100

^a^Reads count.

^b^Percentage of reads.

^c^Mean of percentages.

The diversity of the lung microbiome of patients with SARS-CoV-2 pneumonia at admission was very similar (not statistically significant difference) to the diversity of the microbiome of the patients of the control group: the mean Simpson index was 0.880 ± 0.023 for SARS-CoV-2 patients and 0.902 ± 0.038 for controls (p = 0.213) and the Shannon index was 3.881 ± 0.301 for SARS-CoV-2 patients and 4.213 ± 0.580 for controls (p = 0.187).

#### Pulmonary microbiome of patients with pneumococcal pneumonia

There were 12 patients with pneumococcal pneumonia included in the study. Five had a positive blood culture (four of them also had a positive urine antigen test). Another six had a positive urine antigen test and the last patient was diagnosed by the growth of the pneumococcus on a tracheal aspirate culture.

Four had a mixed pneumonia caused by Influenza virus AH1 and *S. pneumoniae*, and one each with Human Metapneumovirus (hMPV) and Respiratory Syncytial Virus (RSV). In the other 6 patients, *S. pneumoniae* was the unique pathogen detected. Because the diversity indexes of the lung microbiome of patients with simple *S. pneumoniae* pneumonia or coinfected with a virus were similar, patients with a pneumococcal pneumonia were studied as a unique group.

The composition of the microbiome of patients with pneumococcal pneumonia showed that Firmicutes (53.1%) and Proteobacteria (36.5%) comprised the majority of all phyla (Table 7). They were mainly represented by the genus *Streptococcus* (43.7%) and *Haemophilus* (18.4%) (Supplementary Table S6). *Haemophilus* was the predominant genus in two of the four patients that had a mixed Influenza AH1 and *S. pneumoniae* pneumonia (69.4% and 66.9%) and in one of the patients with pure pneumococcal pneumonia (83.4%).

**Table 7:** Respiratory microbiome composition at the phylum level of patients with severe pneumococcal pneumonia.

Patient	SPN-1		SPN-2		SPN-3		SPN-4		SPN-5		SPN-6		SPN-7		SPN-8		SPN-9		SPN-10		SPN-11		SPN-12		Mean
Concomitant viral pathogen	None		RSV		None		Influenza AH1		hMPV		None		None		None		Influenza AH1		Influenza AH1		Influenza AH1		None		
Phylum	Reads^a^	%^b^	Reads	%	Reads	%	Reads	%	Reads	%	Reads	%	Count	%	Count	%	Count	%	Count	%	Count	%	Count	%	%
Actinobacteria	516	0.8	2859	4.0	4621	14.4	223	0.2	857	0.5	1335	3.0	9737	12.6	681	0.2	11	0.0	14	0.1			347	0.5	3.0
Bacteroidetes			19,260	26.9	3429	10.7			877	0.5	490	1.1	12,830	16.6			1834	1.3	1197	9.8			27	0.0	5.6
Chloroflexi													251	0.3											0.0
Crenarchaeota					321	1.0																			0.1
Cyanobacteria					3196	10.0	109	0.1			98	0.2	794	1.0											0.9
Deinococcus-Thermus					10	0.0					314	0.7													0.1
Firmicutes	67,114	99.2	43,870	61.3	9175	28.6	10,061	9.1	181,097	97.2	37,247	83.9	18,844	24.3	34,156	10.9	133,249	97.1	10,888	89.3	1447	3.2	22,453	33.4	53.1
Fusobacteria			3011	4.2	40	0.1			20	0.0			858	1.1	28	0.0	229	0.2							0.5
Gemmatimonadetes													65	0.1											0.0
Proteobacteria	43	0.1	2618	3.7	11,309	35.2	100,208	90.6	3414	1.8	4925	11.1	32,574	42.0	278,693	88.9	1891	1.4	96	0.8	43,143	96.8	44,491	66.1	36.5
Spirochaetes													1,114	1.4											0.1
Synergistetes													237	0.3											0.0
Tenericutes													159	0.2											0.0
Verrucomicrobia													35	0.0											0.0
Total	67,673	100	71,618	100	32,101	100	110,601	100	186,265	100	44,409	100	77,498	100	313,558	100	137,214	100	12,195	100	44,590	100	67,318	100	100

^a^Reads count.

^b^Percentage of reads.

^c^Mean of reads.

The diversity of the lung microbiome of patients with pneumococcal pneumonia at admission was lower than in the control group and in patients with viral pneumonia. The mean Simpson index was 0.369 (range 0.022–0.920) compared to 0.902 (range 0.840–0.947) of controls (p = 0.002) or 0.798 (range 0.651–0.929) for patients with pure influenza pneumonia (p = 0.009) and 0.843 (0.532–0.909) of patients with SARS-CoV-2 pneumonia (p < 0.001).

The Shannon index was 1.417 (range 0.1–4.185) for patients with pneumococcal pneumonia, statically lower the diversity Shannon index for of the patients of the control group (4.214, p < 0.001) and for patients with influenza (3.126, p = 0.027) or SARS-CoV-2 pneumonia (3.882, p < 0.001).

### Utility of mNGS for etiological diagnosis of non-pneumococcal bacterial pneumonia

The BAL of 7 patients admitted to the ICU with radiologically confirmed pneumonia in whom bacterial pneumonia had been diagnosed using conventional methods (antigen detection, culture, PCR detection) were selected according to their bacterial aetiology to study the possibilities of using the mNGS with diagnostic purposes. Unlike the rest of the patients of this work, conventional BALs were collected in these patients using a non-protected catheter.

Two of them had a positive *Legionella pneumophila* urinary antigen test. One of them also had a positive PCR on a tracheal aspirate in which later grew a serogroup 1 *L. pneumophila*. The microbiome of the two patients was almost entirely composed of the pathogenic bacteria: 92.3%, and 89.5% at the species level and 95.4%, and 99.9% at the genus level (Supplementary Table S7).

In the BAL of another patient grew a *Streptococcus pyogenes* in pure culture. In the same BAL an Influenza AH3 virus was detected by RT-qPCR. The microbiome of this patient was also nearly fully composed of the pathogenic bacteria: 79.1% and 99.9%, at the species and genus level, respectively.

Another two patients had a *Haemophilus influenzae* pneumonia. In the BAL and in a tracheal aspirate of the first case, a 66 year old man, a *H. influenzae* grew in pure culture. He also had a RSV detected by RT-qPCR in a pharyngeal exudate. The other, a 60 year old woman, had an *H. influenzae* cultured from a tracheal aspirate. In both patients, the blood-cultures, the *Legionella* and *S. pneumoniae* urine antigens and the qPCR to detect other respiratory viruses and atypical bacterial pneumonia were negative. In the first case, *Haemophilus* represented 90.4% of the genus detected (*H. influenzae* 7.8%, *Haemophilus aegyptius* 48.2% at the species level) and in the second, *Haemophilus* represented 89.2% of the genus detected (*H. influenzae* 7.7%, *Haemophilus aegyptius* 50.8% at the species level).

A 41 years old man was admitted in the ICU due to Leptospirosis, diagnosed by PCR in the urine and blood, and a positive serology. In the BAL of this patient, the genus *Leptospira* represented 26% of the mapped reads. At the species level, *Leptospira interrogans* represented 6.5% of the reads. It was the only respiratory sample in which a *Leptospira* was found.

Finally, a patient with two days of purulent expectoration was admitted to the ICU after a respiratory arrest in the emergency room. Influenza A virus was detected in a pharyngeal swab collected at admission but in the BAL obtained they next day *Klebsiella pneumoniae* grew in significant number (5,000 cfu/mL). At the phylum level, the microbiome showed a 100% of Protobacteria, 42% of *Klebsiella* at the genus level (19.8% *K. pneumoniae* and 21.2% *Klebsiella variicola* at the species level).

## Discussion

Since the existence of a pulmonary microbiome was demonstrated, numerous works have analysed and reviewed different aspects of its composition in relation to respiratory diseases^6,11^. Most of these works have focused on chronic pulmonary diseases^15^, such as COPD^16^, cystic fibrosis^17^, asthma^18^, or bronchiectasis^19^. However, the role of the microbiome in acute pulmonary diseases such as CAP is less well understood, probably due to the difficulty of finding non-antibiotic treated intubated patients in whom a lower respiratory tract sample can be more easily obtained. It is well known the effect of antibiotics in pulmonary microbiota^17,20^.

First, we validated the use of a nested-PCR to detect the lung microbiome due to the low bacterial load in the lungs of some patients, especially when collected with protected catheter to avoid contamination with microbiota of the upper-respiratory tract^21^. Our results showed that there were nearly no differences in the distribution and richness of the most frequent genus within the same sample, showing that nested PCR can improve the results of the number of samples being processed. Due to the difficulty in selecting patients for studies of the lung microbiome, considering the need of intubation, absence of antibiotic treatment, etc., nested PCR can improve the number of patients studied without biasing the composition of the lung microbiome^16,22^.

The pulmonary microbiome of the control group corresponded to that of patients who had undergone elective digestive surgery. Given the impossibility of obtaining mini-BAL samples in healthy people for ethical reasons, we used as control this group of patients that, although not healthy, did not had respiratory pathology. Even so, we could not rule out that the respiratory microbiome of some of them was altered due to the microaspirations of an altered oropharyngeal microbiota^8^.

As in other studies^23^, the pulmonary microbiome of the controls showed a high alpha-diversity, with a predominance of Proteobacteria, Firmicutes and Actinobacteria at the phylum level. Twenty-one genera had between 1 and 8% of all assigned reads, demonstrating the great diversity of the lung microbiome in absence of respiratory pathology. Typical genus of the lung as *Streptococcus*, *Prevotella* and to a lesser extent *Veillonella* were included among these genus, as showed in other studies^6,11^.

In patients with influenza A virus pneumonia alpha-diversity values were similar to those of the microbiome of the control group. This was also observed in patients with other viral infections (Rhinovirus and RSV). As summarized in a recent work, no consistent changes were demonstrated in the diversity of the upper respiratory tract microbiome in virally infected patients and healthy controls in cross-sectional studies^24^. Also, in a mouse model of influenza virus infection, it was shown that the lower respiratory tract is very stable against viral infection, which produces only minor qualitative changes in the composition of its microbiota^25^. The fact that these patients were at early stages of pure Influenza AH1 virus pneumonia (no bacterial coinfection) could be the reason why the changes in their lung bacteriome was nearly inapparent.

Patients with SARS-CoV-2 infection also did not show a differentiate lung microbiome at ICU admission. *Microbacterium*, *Streptococcus*, *Acinetobacter* and *Propionibacterium* were the most frequent genera found. The changes of microbial diversity in patients with SARS-CoV-2 pneumonia is controversial: some studies found a higher diversity (Shannon index) among SARS-CoV-2 patients compared to controls although microbial composition was different^26^. In the upper respiratory tract of patients with SARS-CoV-2 pneumonia it has also been observed a decrease diversity in the microbiome with increasing length of stay in ICU^27^. Other studies, however, have found dysbiosis in the lungs of patients with SARS-CoV-2 pneumonia although in these patients the use of antibiotics was not ruled out^28^.

The lung microbiome of patients with pneumococcal pneumonia showed a lower diversity than the microbiome of controls and of patients with viral pneumonia, with a clear decrease in Actinobacteria and the predominance of two phyla, Firmicutes and Proteobacteria, mainly represented by the genera *Streptococcus* and *Haemophilus*, respectively. The lower diversity observed in the microbiome of patients with pneumococcal pneumonia seems to be due to an increase in the number of pathogenic bacteria, which could also compete with commensal microbiota. The mini-BAL of two patients with pneumonia of unknown origin was also studied. All microbiological test (blood cultures, *Legionella* and pneumococcal urine antigens, viral and atypical bacterial PCRs, etc.) were negative. Both patients had a predominance of Firmicutes (68.9% and 72.1%) mainly *Streptococcus* (59.6% and 51.2%, respectively) (Supplementary Table S8). However, the aetiology of *S. pneumoniae* as responsible of the pneumonia could not be proved.

The number of studies on the lung microbiome in bacterial CAP are scarce, mainly focused in sputum samples that can be contaminated with upper respiratory tract microbiota hindering the interpretation of the results. In an study performed on canine bacterial pneumonia it was observed, as well as in ours, that the relative diversity of bacterial communities was decreased in dogs with bacterial pneumonia^29^. In critically ill patients it was observed a worse clinical outcome (ventilator free days) associated with increased lung bacterial burden and lung enrichment with gut-associated bacteria^30^. Moreover, half of the pneumococcal pneumonias of this study were concomitant to viral infections, specially to influenza AH1 virus, which makes it even more difficult to establish pathogen-microbiome associations. The finding of three patients with pneumococcal pneumonia and a predominance of *Haemophilus* in the metagenomic analysis is not surprising, as mixed CAP are not infrequent, specially the combination of *S. pneumoniae* and *H. influenzae*^31^. Both bacteria habit the same respiratory mucosal environment although the cooperative or competitive nature of this relationship has yet to be determined^32^.

The utility of mNGS to determine the aetiology of bacterial CAP was not specifically the main objective of this study. However, the availability of samples from patients with severe pneumonia caused by different bacterial species prompted us to do so. In the BAL of all of these patients, the pathologic agent was detected, including two cases of *L. pneumophila* and one of *Leptospira* culture-negative respiratory samples. Some studies have found discrepancies between bacterial culture and molecular testing results in patients with pneumonia^33,34^. Molecular techniques appear to be more sensitive than culture for bacterial detection^5,34^, however more studies are needed to show the real potential of metagenomics in the diagnosis of pneumonia.

This study has several limitations. The number of patients included in each type of pneumonia (viral or bacterial) was low, which limits the representativeness of the results. Some samples showed a very low bacterial load and had to be studied after a nested PCR. Although we proved in several samples to have similar results in standard and nested PCRs, small alterations in the microbiome composition after two amplification processes could not be completely ruled out. Another limitation was that only the bacterial microbiome (bacteriome) was studied. The lower alpha diversity observed in bacterial pneumonias was a consequence of the increase in the etiologic pathogen that unbalanced the resident microbiota. In viral pneumonias we could not rule out a decrease in the virome diversity and the fungome (mycobiome) was not studied in any sample. Techniques capable of studying the microbiome as a whole are needed to determine alterations in all the microbes of the lung microbiome. Finally, although a limit of 48 h of antibiotic treatment was admitted as not to cause detectable alterations in the metagenomics study of the lung microbiome, some of the patients had already been treated, what could have altered the microbiome composition. Other studies have demonstrated that bacterial DNA can be detected unaltered by molecular methods even after completed antibiotic treatment^35^.

In conclusion, most of the aetiologic diagnosis of patients admitted to ICU with severe CAP were done applying extensive microbiological diagnostic methods (culture, antigen detection, molecular testing). In patients with viral pneumonia, lung microbiome diversity and composition was similar to that of the microbiome of controls. On the other hand, patients with pneumococcal pneumonia showed a lower microbiome diversity. Further studies including more patients are needed to assess the utility of metagenomic analysis as a complementary tool in the diagnosis of bacterial pneumonia.

### Supplementary Information

Supplementary Table S1.

Supplementary Table S2.

Supplementary Table S3.

Supplementary Table S4.

Supplementary Table S5.

Supplementary Table S6.

Supplementary Table S7.

Supplementary Table S8.

## Data Availability

All raw sequencing data is available as supplementary material.
