# Author Correction: Lung microbiome on admission in critically ill patients with acute bacterial and viral pneumonia

**DOI:** 10.1038/s41598-024-53030-2

**Published:** 2024-02-01

**Authors:** Jose María Marimón, Ane Sorarrain, Maria Ercibengoa, Nekane Azcue, Marta Alonso, Loreto Vidaur

**Affiliations:** 1 Biodonostia, Infectious Diseases Area, Respiratory Infection and Antimicrobial Resistance Group, Microbiology Department, Osakidetza Basque Health Service, Donostialdea Integrated Health Organization, 20014 Donostia-San Sebastian, Spain; 2 Microbiology Department, Donostia University Hospital, 20014 Donostia-San Sebastián, Spain; 3 Intensive Care Unit, Donostia University Hospital, 20014 Donostia-San Sebastián, Spain; 4 Centro de Investigacion Biomedica en Red de Enfermedades Respiratorias (CIBERES), Instituto de Salud Carlos III, Madrid, Spain

Correction to: *Scientific Reports* 10.1038/s41598-023-45007-4, published online 18 October 2023.

The original version of this Article contained an error in the spelling of the author Ane Sorarrain, which was incorrectly given as Ane Sorrarain.

The original Article has been corrected.

